# Standardized minimal acupuncture, individualized acupuncture, and no acupuncture for infantile colic: study protocol for a multicenter randomized controlled trial - ACU-COL

**DOI:** 10.1186/s12906-015-0850-x

**Published:** 2015-09-14

**Authors:** Kajsa Landgren, Iren Tiberg, Inger Hallström

**Affiliations:** Department of Health Science, Faculty of Medicine, Lund University, P.O. Box 157, SE-22100 Lund, Sweden

## Abstract

**Background:**

Despite weak evidence, the use of acupuncture has increased in infantile colic. The only three randomized trials conducted evaluated standardized minimal acupuncture in one single point. Two showed effect but one did not so further research is necessary.

The aims of the study are 1) to test if results in earlier trials conducted in private acupuncture clinics can be repeated at Child Health Centers (CHC) and 2) to compare the effect of two types of acupuncture and no acupuncture in infants with colic at CHC.

**Methods/design:**

a multicenter randomized controlled three-armed trial for infantile colic conducted in four regions of Sweden. Alongside the standard program at their regular Child Health Center infants visit a study center twice a week for 2 weeks. The infants are randomly allocated into three groups.

According to the power analysis, 144 otherwise healthy infants aged 2–9 weeks old, who – according to parents’ registration in a diary – are crying and/or fussing more than three hours per day, more than 3 days per week will be included. Parents register daily in the diary during the baseline week, two intervention weeks, and one more week directly after the last study visit.

At four study visits at the Child Health Center parents meet a nurse for 20–30 min to receive advice and support. The nurse and the parents are blinded for group allocation. Infants are carried to another room, where they spend five minutes with an acupuncturist. Infants randomized to group A receive standardized minimal acupuncture in LI4. Group B receive individualized acupuncture where, according to symptoms, the acupuncturist can choose between the points Sifeng, LI4, and ST36. Group C receives no acupuncture.

The primary outcome is relative difference in crying, counted in minutes. Secondary outcomes are number of infants fulfilling the criteria for colic, and changes in sleep and stooling frequency. Adverse events and blinding are recorded.

Recruitment started in January 2013. During the first 14 months 93 patients were included. Data collection continues until May 2015. No interim analyses have been conducted.

**Discussion:**

The study will provide information about the efficacy and safety of acupuncture as a complement to usual care in infants with colic.

**Trial registration:**

December 29, 2012: ClinicalTrials.gov NCT01761331

## Background

Infantile colic, defined as crying and/or fussing for more than three hours per day, more than 3 days per week, affects 10–20 % of newborns [1] and thus is a relatively common problem. Both infants and parents suffer, and there is a risk of delayed early attachment [2, 3]. Eliminating cow’s milk protein resolves the colic in 20 % of afflicted infants. Currently, no other conventional treatment exists that is both safe and effective, and the use of complementary medicine is increasing. Acupuncture for infantile colic is used in many countries [4] despite weak evidence. The Swedish Council for Health Technology Assessment (SBU) suggests that patients may benefit from acupuncture [5], although it is rarely provided within the Swedish public health system. To this date no serious side effects have been reported.

It is plausible that acupuncture can have effect in the treatment of colic, as acupuncture releases different neurotransmitters and hormones, has both calming and analgesic effects, and affects digestion [6]. The only three published randomized controlled trials were conducted in private clinics and evaluated standardized minimal acupuncture in one single point. Two of the trials used the acupuncture points Large Intestine 4 (LI4) [7, 8] and report a small but significant reduction in crying in the acupuncture groups compared to the control groups. In one of them [8], the needle was stimulated for 20 s; in the other, for only two seconds [7]. A third RCT evaluating the effect of the acupuncture for 30 s in the acupuncture point Stomach 36 (ST36) found no significant difference between groups [9].

Traditional Chinese Medicine (TCM) is a complex and diverse form of therapy, encompassing several styles of acupuncture, among other medical practices. In clinical practice, most TCM acupuncturists differentiate between syndromes and select acupuncture points and needle techniques according to a diagnosis. Diagnoses can, for example, be based on the patient’s pulse, constitution, and signs of “heat” or “cold” [4]. There is a discussion whether the effect of acupuncture depends on the points used or not [10, 11]. TCM acupuncturists claim that point specificity and the choice of points according to patients’ symptoms and signs are vital [4]. For this reason, TCM practitioners select points at each treatment session to match each patient’s most current symptoms. This means that in clinical practice, the same point is rarely chosen for all patients with a specific condition, such as colic, and treatment is rarely given in one single point [4]. That is to say, the trials conducted so far might not reflect clinical practice. Further research is warranted, and trials evaluating clinical practice should be conducted.

In earlier trials conducted at private acupuncture clinics, minimal acupuncture in LI4 reduced the crying in infants with colic. If acupuncture to infants is to be implemented in the public health care it should be given at CHC. The aims of the study are 1) to test if results in earlier trials conducted in private acupuncture clinics can be repeated at Child Health Centers (CHC) and 2) to compare the effect of two types of acupuncture and no acupuncture in infants with colic at CHC.

## Methods

### Design

A multicenter randomized controlled trial, called ACU-COL, started in January 2013 and will collect data until May 2015. ACU-COL is performed with three parallel arms at Child Health Centers (CHC) in four regions of Sweden, and measures the effect of acupuncture in reducing symptoms in infants with colic. Two of the four study centers are located outside the second and third biggest cities in Sweden and the remaining two in smaller cities. Two types of acupuncture (standardized minimal acupuncture in LI4 and individualized acupuncture) are compared to an untreated group.

### Participants

Otherwise healthy infants not more than 8 weeks at registration and not more than 9 weeks when the intervention begins.

Inclusion criteria: infants who, according to parents’ record in the diary, cry and/or fuss more than three hours per day, more than 3 days per week were included and randomized. Infants should have tried a diet excluding cow’s milk protein for at least 5 days.

Exclusion criteria: being born before week 37, not gaining weight properly, having tried acupuncture, or taking any kind of medication (apart from dimeticone or lactobacillus reuteri).

### Participant enrollment

Information about ACU-COL was given to CHC nurses within about 80-km radius of each study center. Parents received information about the trial from nurses and physicians at their ordinary CHC or web-based information via a website (www.spädbarnskolik.se).

Parents who wanted to participate in the trial contacted the project leader (KL) for further information. The project leader checked that the infant was suitable for the trial (Form A) and instructed parents to register the infant’s fussing, crying, colicky crying, sleeping, feeding, and stooling daily in a validated diary (Form B) during a baseline week used for selection. Infants who according to the diary had cried/fussed more than three hours/day more than 3 days and thereby had fulfilled the criteria for colic were included and randomized provided that the parents signed informed consent.

### Randomization

Infants were randomly allocated into one of three groups in proportions of 1:1:1, following randomization lists performed by an independent center for clinical research and development (R&D, Region Skane). The infants were randomized in two strata: infants aged 2–5 weeks and infants aged 6–9 weeks and therefore two randomization lists were created for each of the four centers. Infants were assigned to the three different treatments by sampling without replacement from a block consisting of, to the investigator, unknown number of labels from each treatment group. Researcher and clinicians had no influence on the allocation of infants.

### Blinding

When an infant was included, the project leader informed a project coordinator, who was in charge of the randomization. The coordinator kept the randomization lists confidential and had no contact with or information about the families. The coordinator sent the study number via e-mail to the nurse at the study center and to the project leader, as well as the study number and group allocation to the acupuncturist. The acupuncturist’s records, including the group allocation, were stored in a locked briefcase that only was available for the acupuncturists. Essentially, only the coordinator and the acupuncturist knew each infant’s group allocation. None of them met the parents or discussed the infants with study nurses.

### Usual care

Parallel to the intervention, all infants followed the standard program at their regular Child Health Center (CHC), which includes a weekly visit to a nurse during the first month of life, every second week the second month, and one visit the third month. At these regular visits, the infants are weighed and measured, and the parents get childcare advice.

### Interventions

Parallel to usual care, study participants visited a study CHC where the intervention was conducted twice a week for 2 weeks. Parents met a nurse for 20–30 min. Parents described their situation, discussed the infant’s symptoms, and got evidence based support and advice. All parents received the information that having a baby with colic can be exhausting, and the nurse attempted to give them hope. The nurse informed parents about normal crying, that a healthy 6 week old infant cries an average of 1.5–2.5 h per day and that colic does not negatively affect the child’s development. Breastfeeding mothers were encouraged to continue to do so.

At each of the four study visits, the study nurse brought the infant to a separate treatment room where another nurse, trained in acupuncture, was alone with the infant for approximately five minutes. The acupuncture nurse treated the baby according to allocation and recorded treatment provided, as well as whether the baby cried and, if so, for how long. Adverse events and how they were dealt with was also recorded (Form E). After five minutes, the acupuncturist called the study nurse, who then carried the baby back to the parents.

Group A: Infants received standardized minimal acupuncture in LI4. One needle was inserted approximately 3 mm deep in the point LI4 on the infant’s hand unilaterally for 2–5 s and then withdrawn.
Group B: Infants received individualized acupuncture and the needles could be retained longer. A guideline for the choice of points was developed based on a previous practitioner survey conducted with experienced acupuncturists specialized in pediatrics [4]. It allowed acupuncturists to choose any combination of the acupuncture points Sifeng, LI4, and ST36, based on the symptoms reported in the diary. Maximum 5 needles were inserted approximately 3 mm. The point Sifeng was considered as four insertions, needled about one second each. Needles in LI4 and ST36 were retained for maximum 30 s each.
Group C: Infants spent five minutes with the acupuncturist in the treatment room. The nurse held the infant’s hand and spoke to him/her in a calm voice.

### Sample size

ACU-COL was designed to compare the effect of two types of acupuncture and no acupuncture in infants with colic. According to the power analysis, conducted by an independent statistician at the center for clinical research and development (R&D, Region Skane) and based on the results in an earlier trial with the primary outcome crying [7], 192 infants will be needed to detect a statistically significant difference between the two acupuncture groups assuming that a clinically relevant difference in crying per day between group A and group B is 30 min which is 11 % in relative difference between the first and the second intervention week compared to the baseline week.

#### Outcome measurement

Primary outcome is relative difference in crying (mean values for the sum of fussing, crying and colicky crying) during the baseline week, during each of the two intervention weeks and during 6 days after the last treatment.

A secondary outcome is how many infants in each group who still cries and/or fusses more than three hours/day more than 3 days per week; i.e. fulfills the criteria for colic, each intervention week. Other secondary outcomes are change in frequency of stooling per day and change in hours of sleep per day during the same time frames. Adverse events, eventual parallel treatment or medication, how much infants cried from being needled and how well parents are blinded will be reported. Points chosen by the acupuncturists for infants in group B will be reported.

### Data collection

Parents register in the diary (Form B) daily during the base line week, two intervention weeks, and 6 days after the last visit at the study CHC. The study nurse collects the diaries at the study visits. The diaries from the follow up week are sent to the project leader by mail.

At the first visit the nurse collects demographic data (Form C) and information about smoking, allergies, colic in the family, complications, and the mother’s intake of prescribed medication during the pregnancy and after the delivery. Information about the infants’ feeding, birth weight, when the excessive crying started, and use of probiotics, dimeticone, herbal treatment, etc. is also collected in Form C.

In Form D parents’ experiences of changes in the infants crying, stooling and sleep as well as their perception of if their infant has received acupuncture or not, and whether they have noted any side effects associated with acupuncture are reported.

The acupuncturist note in a Form E which of the points that was used, time for stimulation, possible crying and if so for how long.

One week after the fourth visit, and after the parents have sent in the diary for the last week parents are phoned and asked the questions in Form D once more. Parents also report eventual treatments or medication provided by the CHC or by other therapists parallel to the study participation (Form F). Thereafter the parents are told their infant’s study allocation.

When each of the instruments is used in the trial is described in Table 1.

**Table 1:** Telephone contacts, visits and instruments

	Baseline week for selection of infants fulfilling the criteria for colic		Intervention week 1		Intervention week 2		Follow up	
Telephone	Form A	Form A						Form F
Parents daily registration in a diary	Form B	Form B	Form B	Form B	Form B	Form B	Form B	
Visits to the study center.			X	X	X	X		
Study nurse collects informed consent			Form H					
Study nurse collects baseline data			Form C					
Study nurse collects data				Form D	Form D	Form D		
Acupuncturist registers treatment			Form E	Form E	Form E	Form E		

#### Statistical analysis

Statistical analyzes will be made from the data collected in forms and records (see Table 1), and carried out following a structured data management plan and by using appropriate statistical methods (dependent upon type and variable) for descriptive data as well as multivariate analysis.

We will analyze usual care plus standardized minimal acupuncture (group A) and individualized acupuncture (group B) versus usual care plus four extra visits to a CHC (group C). The mean difference over time and between groups in reduction in % of colicky crying, crying and fussing in minutes/day will be compared using relevant statistical methods depending on distribution.

We will use analysis of covariance at the baseline week, the first intervention week, the second intervention week and the week after the last visit to the study center to assess whether clinical differences, if any, are significant.

How many infants in each group who still cries and/or fusses more than three hours/day more than 3 days per week, i.e. fulfills the criteria for colic, each intervention week will be assessed by the difference in survival curves (days to crying <3 h/day) using Kaplan–Meier analysis.

Additional descriptive measurements will be presented on back ground data, the acupuncturists’ choice of points for group B, results on separate study centers and on blinding.

All data in this trial will be assessed by SPSS v22.0 (SPSS, Chicago, IL, USA). P <0.05 will be considered statistically significant.

### Ethical considerations

Research with children requires careful ethical reflection. Infants in our study are vulnerable and they can not sign the informed consent themselves. Infants with colic suffer and it is important to involve them in research to be able to find effective treatments. In this trial the infants were separated from the parents for five minutes for blinding reasons. Parents were informed about this and could choose not to participate. The separation was done as gently as possible, and when the parent and the infant were ready. For example the mother could breastfeed before leaving the baby to the nurse. The Regional Ethical Review Board in Lund and the Department Manager at each center approved the trial (Dnr 2012/620).

## Discussion

This study will provide information about the efficacy of acupuncture as a complement to usual care and increase the current knowledge about safety in acupuncture with infants.

To increase validity, infants’ crying is measured in a detailed diary, we follow a strict protocol and we measure how well parents are blinded. The results will be published regardless of outcome in an article following the STRICTA guidelines.

### Trial status

Recruitment began in January 2013. During the first 14 months, 93 infants were included. Data collection will continue at least until May 2015. No interim analyses have been conducted. Analysis of data is expected to be complete during Autumn 2015, followed by submission to a peer-reviewed journal in December 2015.

## Abbreviations

CHC: Child health center
RCT: Randomized controlled trial
SBU: Swedish council on health technology assessment
TCM: Traditional Chinese medicine
